# Effects of the Photosystem II Inhibitors CCCP and DCMU on Hydrogen Production by the Unicellular Halotolerant Cyanobacterium* Aphanothece halophytica*

**DOI:** 10.1155/2019/1030236

**Published:** 2019-06-27

**Authors:** Sunisa Pansook, Aran Incharoensakdi, Saranya Phunpruch

**Affiliations:** ^1^Department of Biology, Faculty of Science, King Mongkut's Institute of Technology Ladkrabang, Bangkok 10520, Thailand; ^2^Laboratory of Cyanobacterial Biotechnology, Department of Biochemistry, Faculty of Science, Chulalongkorn University, Bangkok 10330, Thailand; ^3^Academy of Science, Royal Society of Thailand, Bangkok 10300, Thailand; ^4^Bioenergy Research Unit, Faculty of Science, King Mongkut's Institute of Technology Ladkrabang, Bangkok 10530, Thailand

## Abstract

The unicellular halotolerant cyanobacterium* Aphanothece halophytica* is a potential dark fermentative producer of molecular hydrogen (H_2_) that produces very little H_2_ under illumination. One factor limiting the H_2_ photoproduction of this cyanobacterium is an inhibition of bidirectional hydrogenase activity by oxygen (O_2_) obtained from splitting water molecules via photosystem II activity. The present study aimed to investigate the effects of the photosystem II inhibitors carbonyl cyanide m-chlorophenyl hydrazone (CCCP) and 3-(3,4-dichlorophenyl)-1,1-dimethylurea (DCMU) on H_2_ production of* A*.* halophytica *under light and dark conditions and on photosynthetic and respiratory activities. The results showed that* A*.* halophytica* treated with CCCP and DCMU produced H_2_ at three to five times the rate of untreated cells, when exposed to light. The highest H_2_ photoproduction rates, 2.26 ± 0.24 and 3.63 ± 0.26  *μ*mol H_2 _g^−1^ dry weight h^−1^, were found in cells treated with 0.5 *μ*M CCCP and 50 *μ*M DCMU, respectively. Without inhibitor treatment,* A*.* halophytica* incubated in the dark showed a significant increase in H_2_ production compared with cells that were incubated in the light. Only CCCP treatment increased H_2_ production of* A*.* halophytica* during dark incubation, because CCCP functions as an uncoupling agent of oxidative phosphorylation. The highest dark fermentative H_2_ production rate of 39.50 ± 2.13  *μ*mol H_2 _g^−1^ dry weight h^−1^ was found in cells treated with 0.5 *μ*M CCCP after 2 h of dark incubation. Under illumination, CCCP and DCMU inhibited chlorophyll fluorescence, resulting in a low level of O_2_, which promoted bidirectional hydrogenase activity in* A*.* halophytica* cells. In addition, only CCCP enhanced the respiration rate, further reducing the O_2_ level. In contrast, DCMU reduced the respiration rate in* A*.* halophytica.*

## 1. Introduction

Molecular hydrogen (H_2_) has attracted a great deal of interest from researchers because H_2_ combustion liberates a high heating value with 141.6 MJ kg^−1^ [1] and does not emit polluting gases to the environment. H_2_ production is a result of many processes, including physical, chemical, and biological processes. Biological H_2_ production can be established in many kinds of microorganisms such as photosynthetic bacteria, fermentative bacteria, green algae, and cyanobacteria [2]. Among these microorganisms, cyanobacteria show high capability because they can generate H_2_ using electrons obtained from a light reaction of the photosynthetic pathway and/or from the degradation of storage carbohydrates within cells in darkness [3, 4].

The unicellular cyanobacterium* Aphanothece halophytica *is a halotolerant microorganism that can grow in a wide range of salinity from 0.25 to 3.0 M NaCl [5].* A. halophytica* produces a large amount of dark fermentative H_2_ compared with other marine cyanobacteria [6, 7]. H_2_ production by* A. halophytica* is catalyzed by bidirectional hydrogenase and occurs particularly under nitrogen-deprived and dark anaerobic conditions [6–8]. Hydrogenase is the only enzyme that catalyzes both H_2_ uptake and H_2_ production in this organism [8]. Due to the high sensitivity of bidirectional hydrogenase to oxygen (O_2_) [9], which is the main product when photosystem II (PSII) activity splits a water molecule, H_2_ production by* A. halophytica* decreases in the light [7]. To enhance H_2_ production by* A. halophytica*, O_2_ must be removed. One way to eliminate the generation of O_2_ from splitting water molecules during photolysis is to use photosystem II inhibitors.

Carbonyl cyanide m-chlorophenyl hydrazone (CCCP) has long been recognized as a photosystem II inhibitor of cyanobacteria and green algae [10]. CCCP has been shown to inhibit the photochemical activity of PSII under illumination in cyanobacteria* Synechocystis* sp. PCC 6803 [11],* Synechococcus *sp. PCC 7942 [12],* Nostoc *sp., and* Lyngbya* sp. [13] and green algae* Chlorella ellipsoidea* [14] and* Platymonas subcordiformis *[15]. This inhibition leads to a decrease in O_2_ production. CCCP can also function as an uncoupling agent of oxidative phosphorylation [16]. It disrupts the proton motive force by releasing protons across the thylakoid membrane, resulting in an inhibition of ATP synthesis. Consequently, a large number of electrons and protons can be transferred to bidirectional hydrogenase to enhance H_2_ production [17]. It has been reported that CCCP increased H_2_ production in the cyanobacteria* Oscillatoria chalybea* and* Synechocystis* sp. PCC 6803 [18] and in the green algae* Chlamydomonas reinhardtii* [19],* P. subcordiformis *[17, 20, 21], and* Platymonas helgolandica* var.* tsingtaoensis* [22]. In addition, this inhibition of ATP synthesis resulted in an increase in the rate of dark respiration in cyanobacteria* Anabaena variabilis *[23] and* Anacystis nidulans *[24] and green alga* C. reinhardtii* [25].

Another PSII inhibitor is 3-(3,4-dichlorophenyl)-1,1-dimethylurea (DCMU) [26]. DCMU can block electron transfer between the primary quinone electron accepter (Q_A_) and secondary quinone electron accepter (Q_B_) on the reducing side of PSII [26]. This interrupts the photosynthetic electron transport chain in photosynthesis and thus reduces the generation of O_2_ from splitting water molecules via PSII. DCMU has been shown to inhibit PSII activity in cyanobacteria* Aphanocapsa* 6308 [27],* Nostoc *sp., and* Lyngbya* sp. [13] and green alga* Scenedesmus quadricauda* [28]. DCMU influences other cellular processes, such as cyclic phosphorylation, chlorophyll synthesis, and fatty acid synthesis [29]. In previous reports, H_2_ production of the cyanobacteria* Anabaena* spp. strains CA and 1F [30],* Anabaena cylindrica *[31],* Anabaena* 7120 [32], and the green alga* P. helgolandica* var.* tsingtaoensis *[22] was increased in the presence of DCMU under illumination. In addition, DCMU caused the inhibition of dark respiration in cyanobacteria* Plectonema boryanum *[33] and* Anabaena flos-aquae *[34].

The goal of the present study was to investigate the effects of the PSII inhibitors CCCP and DCMU on H_2_ production by the cyanobacterium* A. halophytica.* The data will improve our understanding of the functional relationships between H_2_ metabolism and photosynthetic and respiration efficiency. The knowledge gained in this study will be useful to enhance H_2_ production by* A. halophytica* under light or dark conditions by a use of the effective PSII inhibitors, DCMU and CCCP. H_2_ evolution by this cyanobacterium might be one of the most promising ways to produce alternative clean energy fuel in the future.

## 2. Materials and Methods

### 2.1. Growth Conditions

*A. halophytica* was cultivated in a 250-mL Erlenmeyer flask containing 100 mL of BG11 medium (pH 7.4) [35] supplemented with Turk Island salt solution [36]. The initial cell concentration was adjusted to an optical density of approximately 0.1 at 730 nm. Cells were shaken at 120 rpm at 30°C under a cool white light intensity of 30 *μ*mol photons m^−2^ s^−1^ for 7 days.

### 2.2. Application of CCCP and DCMU

After the 7 days of growth, 100 mL of* A. halophytica* cells was harvested by centrifugation at 8,000 x* g* at 4°C for 10 min. The cell pellet was washed twice and resuspended in 100 mL of nitrogen-deprived BG11 (BG11_0_) supplemented with Turk Island salt solution. The cells in suspension were transferred to a 250-mL Erlenmeyer flask and incubated on a rotary shaker at 120 rpm at 30°C under 30 *μ*mol photons m^−2^ s^−1^ for 24 h. Cells were subsequently harvested by centrifugation and resuspended in 5 mL of BG11_0_ supplemented with Turk Island salt solution. Next, a 5-mL volume of the cells in suspension was transferred to a 10-mL glass vial. CCCP and DCMU were subsequently added into the cell suspension with final concentrations of 0–5 *μ*M and 0–250 *μ*M, respectively. The vials were sealed with a rubber stopper with an aluminum rim and incubated at 30°C under 30 *μ*mol photons m^−2^ s^−1^ for 2 h. The vials were subsequently purged with argon gas for 10 min to establish anaerobic conditions. The vials were further incubated under light at 30°C. Aliquots of cells in suspension after incubation for 2, 24, 48, 72, and 96 h were collected for analysis of cell and chlorophyll concentrations. Bidirectional hydrogenase activity, photosynthetic efficiency of PSII by chlorophyll fluorescence measurement, and dark respiration rate were analyzed after the cells were exposed to the light for 2 h. H_2_ production was analyzed after the cells were placed in both light and dark conditions for 2 h.

### 2.3. Measurement of Cell Concentration and Chlorophyll-a Concentration

The cell concentration of* A. halophytica* was analyzed using a hemocytometer under a microscope (Nikon Eclipse Ci-L, Japan). To analyze the chlorophyll-a concentration, 1 mL of a cell culture was harvested by centrifugation at 8,000 x* g* at 4°C for 10 min. A 1-mL volume of 90% (v/v) methanol was added to the cell pellet and mixed by vortexing. The mixture was incubated at 25°C in the dark for 1 h. The chlorophyll-a content was determined by measuring the absorbance of the extract at 665 nm by spectrophotometer [37].

### 2.4. Measurement of H_2_ Production

H_2_ concentration in 500 *μ*L of headspace gas was analyzed by gas chromatograph (Hewlett-Packard HP5890A, Japan) with a molecular sieve 5°A, 60/80 mesh packed column using a thermal conductivity detector under previously described conditions [6]. The H_2_ production rate was calculated as a term of *μ*mol H_2 _g^−1^ dry weight h^−1^.

### 2.5. Measurement of Bidirectional Hydrogenase Activity

The bidirectional hydrogenase activity in the* A. halophytica* sample was determined after cells were incubated with various concentrations of CCCP and DCMU under the light for 2 h. Bidirectional hydrogenase activity was measured in the presence of dithionite-reduced methyl viologen. The assay contained 1 mL of cells in suspension and 1 mL of 25 mM phosphate buffer (pH 7.0) containing 2.5 mM methyl viologen and 10 mM sodium dithionite [7]. The reaction mixture was incubated under dark anaerobic conditions at 25°C for 15 min before H_2_ production was measured using gas chromatography, under previously described conditions [7]. Bidirectional hydrogenase activity was calculated in terms of *μ*mol H_2 _g^−1^ dry weight min^−1^.

### 2.6. Dark Respiration Rate Measurement

The dark respiration rate was monitored at 25°C using a Clark-type oxygen electrode (Hansatech, UK). First, 2 mL of cells in suspension was added to the chamber and illuminated under 300 *μ*mol photons m^−2^ s^−1^ of white light until the O_2_ concentration was constant. Then, the respiratory rate was measured as O_2_ consumption in the dark for 15 min. The dark respiration rate was calculated as a term of *μ*mol O_2 _g^−1^ dry weight min^−1^.

### 2.7. Fluorescence Emission Spectra Measurement

Chlorophyll fluorescence emission spectra were determined at room temperature by spectrofluorometer (Jasco, Model FP-6300, Japan). First, 1 mL of cyanobacteria treated and not treated with CCCP and DCMU was exposed to light at 2,000 *μ*mol photons m^−2^ s^−1^ at room temperature for 10 min prior to chlorophyll fluorescence measurement, following Joshua et al. [38]. The chlorophyll fluorescence measurement was carried out using the excitation wavelength at 437 nm.

### 2.8. Statistical Data Analysis

The data in this study were statistically compared using a one-way ANOVA with Duncan's post hoc test. Differences between means were considered significant at 0.05 (*p* < 0.05). Data were analyzed using IBM SPSS statistic 23 (IBM Corp., USA).

## 3. Results

### 3.1. Effects of CCCP and DCMU on Cell and Chlorophyll-a Concentrations under N-Deprivation

**Figure 1:**
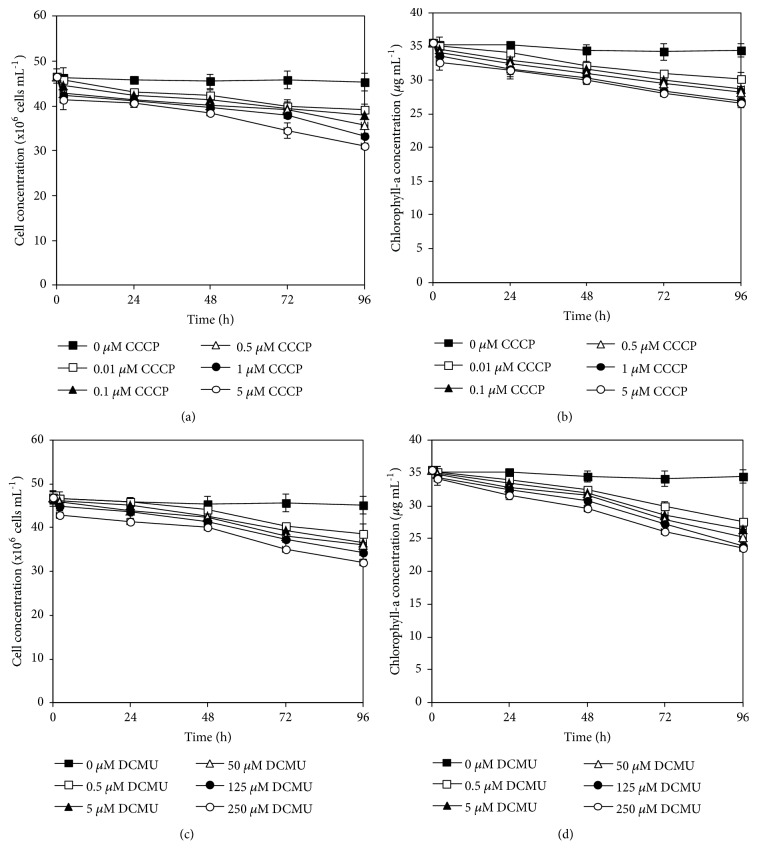
Effects of various concentrations of carbonyl cyanide m-chlorophenyl hydrazone (CCCP) and 3-(3,4-dichlorophenyl)-1,1-dimethylurea (DCMU) on cell concentrations (a, c) and chlorophyll-a concentrations (b, d) of* Aphanothece halophytica *after various incubation durations.

After* A. halophytica* cells were incubated in a nitrogen-deprived medium containing various concentrations of CCCP (0–5 *μ*M) and DCMU (0–250 *μ*M) under light for 2, 24, 48, 72, and 96 h, cell and chlorophyll-a concentrations were measured. The concentrations of both decreased after the cells were incubated in BG11_0_ containing CCCP or DCMU. Both concentrations slightly decreased in the first 2 h of incubation and continued to decrease to 96 h of incubation (Figure 1). Higher concentrations and longer incubation times of CCCP and DCMU led to an obvious decrease in the cell and chlorophyll-a concentrations of* A. halophytica *(Figure 1).

### 3.2. Effects of CCCP and DCMU on H_2_ Production

**Figure 2:**
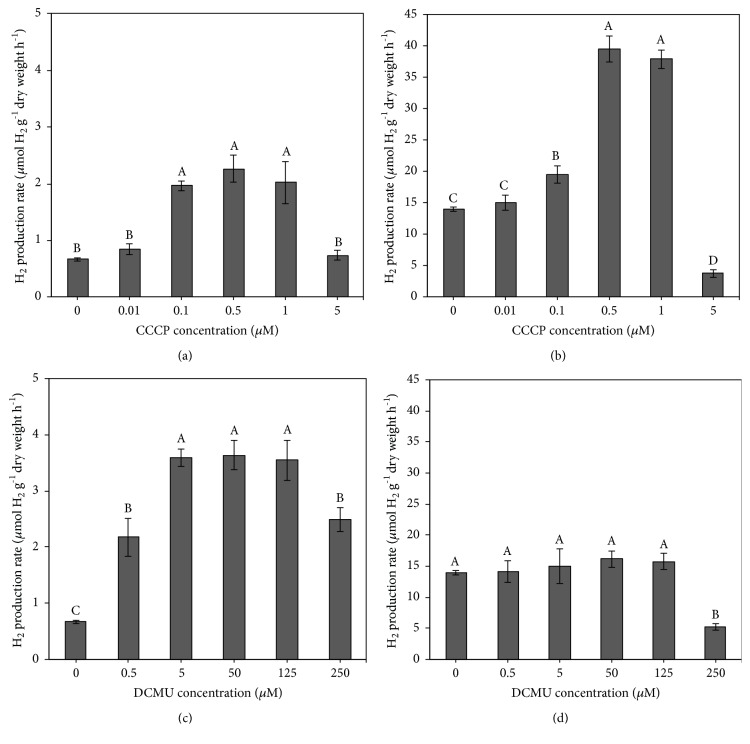
Effects of various concentrations of carbonyl cyanide m-chlorophenyl hydrazone (CCCP) and 3-(3,4-dichlorophenyl)-1,1-dimethylurea (DCMU) on H_2_ production rate by* Aphanothece halophytica* after 2 hours of incubation under the light (a, c) and under darkness (b, d). Data are means ± SD (n = 3). Different letters above columns indicate a significant difference according to Duncan's multiple range test at* p* < 0.05.

*A. halophytica* cells were treated with various concentrations of CCCP (0–5 *μ*M) and DCMU (0–250 *μ*M) and incubated under light at 30°C for 2 h before H_2_ was measured under dark and light anaerobic conditions. The cells that were incubated in the dark, with or without CCCP and DCMU treatment, generated H_2_ at a higher rate than did those incubated under the light (Figure 2). For cells treated with CCCP, the H_2_ production rates, under conditions of both illumination and darkness, were significantly increased, corresponding with the higher concentrations of CCCP (Figures 2(a) and 2(b)). However, the highest concentration of CCCP (5 *μ*M) resulted in the lowest H_2_ production rates (Figures 2(a) and 2(b)). The highest H_2_ production rates of 2.26  ±  0.24 and 39.50  ±  2.13  *μ*mol H_2 _g^−1^ dry weight h^−1^ were found in* A. halophytica* cells treated with 0.5 *μ*M CCCP under light and dark conditions (Figures 2(a) and 2(b)). These H_2_ production rates were approximately threefold higher than those of cells without CCCP treatment.

In the presence of DCMU,* A. halophytica* showed a higher H_2_ production rate only when cells were incubated under light (Figure 2(c)). Dark fermentative H_2_ production was not increased in cells treated with all concentrations of DCMU (Figure 2(d)). Interestingly, in the presence of 250 *μ*M DCMU, dark fermentative H_2_ production was obviously decreased and was lower than that in cells without DCMU treatment (Figure 2(d)). The highest H_2_ production rates of 3.63  ±  0.26 and 16.19  ±  1.32  *μ*mol H_2 _g^−1^ dry weight h^−1^ were found in* A. halophytica* cells treated with 50 *μ*M DCMU under light and dark conditions (Figures 2(c) and 2(d)). The results indicated that CCCP increased the H_2_ production rate under light and dark conditions and that DCMU increased it only under the light.

### 3.3. Effects of CCCP and DCMU on Bidirectional Hydrogenase Activity

**Figure 3:**
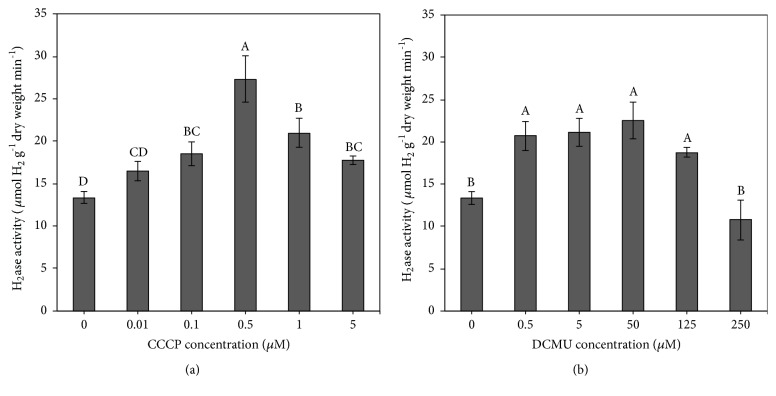
Effects of various concentrations of carbonyl cyanide m-chlorophenyl hydrazone (CCCP) (a) and 3-(3,4-dichlorophenyl)-1,1-dimethylurea (DCMU) (b) on bidirectional hydrogenase of* Aphanothece halophytica* after 2 hours of treatment under illumination. Data are means ± SD (n = 3). Different letters above columns indicate a significant difference according to Duncan's multiple range test at* p* < 0.05.

To determine whether an increase in H_2_ production after CCCP and DCMU treatment resulted from increased bidirectional hydrogenase activity,* A. halophytica* cells were treated or not treated with CCCP or DCMU and incubated under the light for 2 h before bidirectional hydrogenase activity was measured. The results showed that bidirectional hydrogenase activity was higher when cells were treated with higher CCCP and DCMU concentrations. The highest bidirectional hydrogenase activity levels of 27.32  ±  2.73 and 22.58  ±  2.15  *μ*mol H_2 _g^−1^ dry weight min^−1^ were found in cells treated with 0.5 *μ*M CCCP and 50 *μ*M DCMU, respectively (Figures 3(a) and 3(b)). When CCCP and DCMU concentrations exceeded these concentrations, the bidirectional hydrogenase activity level decreased (Figures 3(a) and 3(b)). As expected, bidirectional hydrogenase activities were related to H_2_ production rates (Figures 2 and 3).

### 3.4. Effect of CCCP and DCMU on Chlorophyll Fluorescence

**Figure 4:**
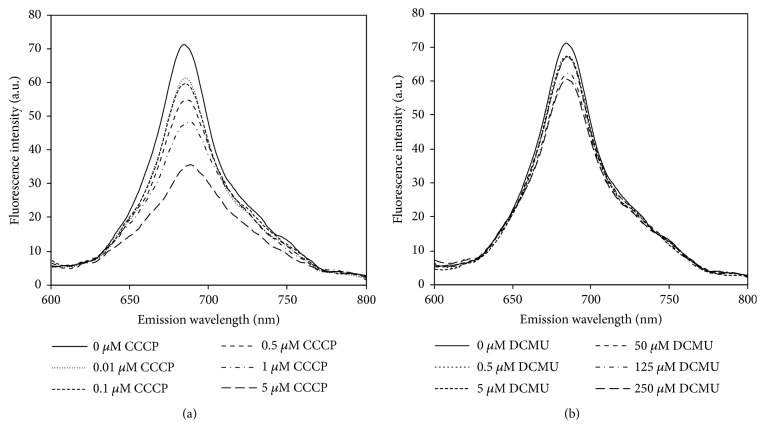
Fluorescence emission spectra of* Aphanothece halophytica *incubated in BG11_0_ (pH 7.4) supplemented with Turk Island salt solution containing various concentrations of carbonyl cyanide m-chlorophenyl hydrazone (CCCP) (a) and 3-(3,4-dichlorophenyl)-1,1-dimethylurea (DCMU) (b) after 2 hours of treatment under illumination.

Chlorophyll fluorescence emission spectra of* A. halophytica* cells treated with various concentrations of CCCP and DCMU under the light for 2 h were measured. The results showed that the chlorophyll fluorescence emission spectra of* A. halophytica* cells treated with higher concentrations of CCCP or DCMU were significantly lower than those that were not treated (Figure 4), suggesting that these inhibitors could inhibit PSII efficiency, leading to a decrease in the O_2_ level in vials (data not shown), an increase in bidirectional hydrogenase activity (Figure 3), and an increase in H_2_ production rate (Figure 2).

### 3.5. Effects of CCCP and DCMU on Dark Respiration

**Figure 5:**
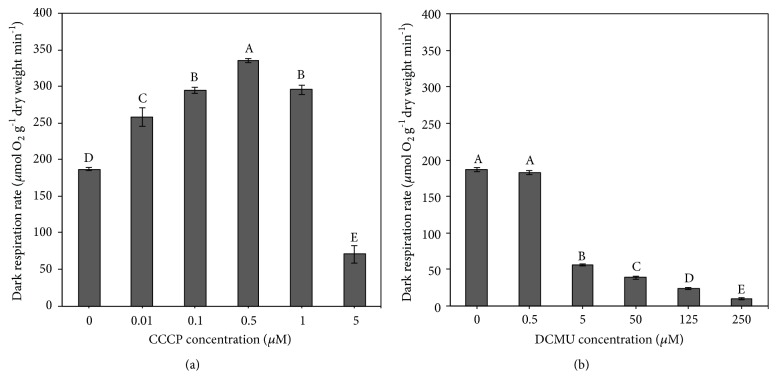
Effects of various concentrations of carbonyl cyanide m-chlorophenyl hydrazone (CCCP) (a) and 3-(3,4-dichlorophenyl)-1,1-dimethylurea (DCMU) (b) on dark respiration rate of* Aphanothece halophytica* after 2 hours of treatment under illumination. Data are means ± SD (n = 3). Different letters on columns indicate a significant difference according to Duncan's multiple range test at* p* < 0.05.

The measurement of dark respiration rate was performed in* A. halophytica* cells after they were treated or not treated with CCCP or DCMU. Cells treated with 0.01–1 *μ*M CCCP showed higher dark respiration rates than those that were not treated with CCCP (Figure 5(a)). The highest dark respiration rate of 335.30  ±  3.32  *μ*mol O_2 _g^−1^ dry weight min^−1^ was found in cells that were treated with 0.5 *μ*M CCCP, and the lowest dark respiration rate was found in cells treated with 5 *μ*M CCCP (Figure 5(a)). DCMU concentrations higher than 0.5 *μ*M reduced the dark respiration rate of* A. halophytica* cells (Figure 5(b)).

## 4. Discussion

### 4.1. Effects of CCCP and DCMU on Cell Inhibition

The treatment of* A. halophytica* cells with CCCP led to a reduction in cell concentration (Figure 1(a)) and chlorophyll-a concentration (Figure 1(b)), especially in cells that received high concentrations of CCCP over long-term incubations (Figures 1(a) and 1(b)). In the absence of CCCP, cells did not show any changes in the cell and chlorophyll-a concentrations. The cell and chlorophyll-a concentrations also did not increase because all cells were incubated in BG11_0_ lacking in NaNO_3_, which is a nitrogen source for cyanobacterial growth. CCCP, which functions as the PSII inhibitor, inhibited the rate of electron flow through the photosynthetic electron transport chain in the thylakoid membrane of cyanobacterial cells [13]. Consequently, cell and chlorophyll concentrations in* A. halophytica* were decreased. These results were consistent with previous studies showing a decrease in the optical density and cell concentration of other microalgae [39, 40]. In* Synechococcus* sp., the optical density at 750 nm and viable cell count were decreased after the addition of 10 *μ*M CCCP [39]. The unicellular green alga* C. reinhardtii* showed MICs at 8.5 and 14.6 *μ*M for CCCP under heterotrophic and photoautotrophic growth conditions, respectively [40]. In case of chlorophyll content, the green algae* P. helgolandica* var.* tsingtaoensis* and* C. reinhardtii* showed a decrease in chlorophyll content when cell cultures were treated with 15 *μ*M CCCP [19, 22].

The treatment of* A. halophytica* with DCMU also caused a reduction in cell concentration (Figure 1(c)) and chlorophyll-a concentration (Figure 1(d)). DCMU, similar to CCCP, is responsible for inhibition of photosynthetic activity; therefore, DCMU treatment inhibited cell growth and chlorophyll concentrations as shown in Figures 1(c) and 1(d). These results agreed with previous studies. In* Synechocystis* sp. PCC 6803, the optical density at 730 nm was decreased in the presence of 0.1 *μ*M DCMU [41]. DCMU at 0.1 *μ*M caused a significant reduction in the growth of colonies of algae* Eudorina elegans* [42] and* Nannochloropsis* [43]. In the presence of DCMU, the chlorophyll concentrations of the N_2_-fixing cyanobacteria* Nostoc* sp. G3 and* A. variabilis* obviously decreased [44, 45]. The results of the present study suggested that high concentrations of CCCP and DCMU and long-term incubation caused cell toxicity and death.

### 4.2. H_2_ Production of* A. halophytica* under Light and Dark Conditions

H_2_ production rates by* A. halophytica* cells incubated in the dark were higher than those incubated under the light, in the presence or absence of inhibitors (Figure 2), indicating that light can inhibit H_2_ production by* A. halophytica. A. halophytica* cells under the light produced more O_2_ than did those in the dark (data not shown) due to the generation of O_2_ from the splitting of water molecules via PSII activity in the thylakoid membrane. O_2_ inhibits the bidirectional hydrogenase activity of* A. halophytica* cells, resulting in lower H_2_ production. In the dark,* A. halophytica* cells had reduced photolysis but engaged in dark respiration, leading to a lower O_2_ concentration in the system and enhanced H_2_ production. Moreover, nitrogen-deprived cells of* A. halophytica* were able to generate more H_2_ from electrons acquired through the degradation of stored glycogen under dark, anaerobic conditions than from photosynthesis under light conditions [6–8].

### 4.3. Effects of CCCP on H_2_ Production, Bidirectional Hydrogenase Activity, Photosynthetic Activity, and Dark Respiration

The CCCP-treated* A. halophytica* showed significantly higher H_2_ production under both light and dark conditions than CCCP-untreated cells. The highest H_2_ production rates of 2.26  ±  0.24 and 39.50  ±  2.13  *μ*mol H_2 _g^−1^ dry weight h^−1^ were found in cultures treated with 0.5 *μ*M CCCP and incubated under light and dark conditions, respectively (Figures 2(a) and 2(b)). These H_2_ production rates were approximately threefold higher than those in the absence of CCCP. CCCP-treated cells also produced less O_2_, as measured in the gas (data not shown). The lower O_2_ concentration in the glass vial caused increased bidirectional hydrogenase activity, as shown in Figure 3(a), and increased the H_2_ production rate (Figure 2). Our results agree with previous studies showing that H_2_ production rates of the cyanobacteria* O. chalybea* and* Synechocystis* sp. PCC 6803 treated with CCCP were higher than those of untreated cells [18]. In green algae, H_2_ production by* C. reinhardtii*,* P. subcordiformis*, and* Tetraselmis subcordiformis* was also increased in CCCP-treated cells [17, 19, 46].

CCCP-treated* A. halophytica* cells showed an increase in the H_2_ production rate under dark conditions (Figure 2(b)). This enhancement most likely was not due to decreased PSII activity by CCCP, but to the inhibition of oxidative phosphorylation by another effect of CCCP as an uncoupler agent [33]. In green algae, CCCP inhibits the flow of electrons in the electron transport chain and promotes the pumping of protons in the oxidative phosphorylation reaction by transporting protons across the thylakoid membrane [20, 47]. As a result, the activity of ATP synthase is reduced, and ATP synthesis is inhibited. The released or excess protons and electrons could be reduced by bidirectional hydrogenase to generate H_2_ [17].

To confirm the effects of CCCP on H_2_ production by* A. halophytica*, bidirectional hydrogenase activity, photosynthetic activity, and dark respiration rate were measured. A treatment of 0.5 *μ*M CCCP produced the highest bidirectional hydrogenase activity level (Figure 3(a)), indicating that concentration of CCCP at 0.5 *μ*M is optimal for promoting bidirectional hydrogenase activity in* A. halophytica*. In a previous study, a treatment with 10 *μ*M CCCP could increase bidirectional hydrogenase activity in* Anabaena siamensis* TISTR 8012 [48]. Therefore, the CCCP concentration influencing hydrogenase activity is species-dependent. However, the chlorophyll fluorescence intensity of* A. halophytica* cells decreased as CCCP concentrations increased (Figure 4(a)). Evidently, CCCP inhibited photosystem II activity, contributing to the lower chlorophyll fluorescence, as shown in Figure 4(a). Moreover, CCCP could inhibit ATP synthesis from working as an uncoupler of oxidative phosphorylation and subsequently increase the respiration rate, as shown in Figure 5(a). The decrease in O_2_ photoevolution, together with the increase in O_2_ consumption, promoted a low level of O_2_ in the system, which is favorable for bidirectional hydrogenase activity. Our results were similar to previous results reported in many cyanobacterial and green algal strains, demonstrating that CCCP reduced PSII photochemical activity [17, 19, 21, 46, 49] and enhanced the rate of dark respiration [23]. It has been reported that the rate of dark respiration was markedly enhanced by addition of 5 and 10 *μ*M CCCP to the cultures of* A. variabilis* [23] and* A. nidulans* [24]. In* C. reinhardtii*, CCCP at 2.5 *μ*M increased the dark respiration rate by 40% without influencing photosynthesis [25]. However, in this study the effect of CCCP on the dark respiration rate in* A. halophytica* was dependent on the CCCP concentration. These data on the stimulation of H_2_ photoevolution and dark fermentative H_2_ production by CCCP treatment may be used to optimize H_2_ production by* A. halophytica *in the future.

### 4.4. Effects of DCMU on H_2_ Production, Bidirectional Hydrogenase Activity, Dark Respiration, and Photosynthetic Activity

DCMU-treated* A. halophytica* produced H_2_ at a significantly higher rate than did DCMU-untreated cells under light conditions (Figure 2(c)) but not under darkness (Figure 2(d)). Evidently, DCMU functioned as a PSII inhibitor in the light, leading to the reduction of O_2_ photoevolution from photolysis. Therefore, the decreased O_2_ level caused an increase of H_2_ production. These results were consistent with the previous results described for CCCP-treated cells. However, under dark conditions, the cyanobacterial cells could not perform photosynthesis and thus were unable to generate O_2_. Therefore, DCMU might not inactivate PSII activity under darkness, resulting in a constant H_2_ production rate compared with the untreated cells. In addition, it is likely that DCMU could not promote dark fermentative H_2_ production by* A. halophytica. *These results contrasted with those of studies in* Synechocystis* sp. PCC 6803, which reported higher H_2_ production in the presence of 75 *μ*M DCMU under dark and anaerobic conditions [50, 51].

Under light conditions, the H_2_ production rate of 50 *μ*M DCMU-treated* A. halophytica* cells was threefold higher than that of untreated cells (Figure 2(c)). This high rate resulted from the highest observed bidirectional hydrogenase activity in the present study, recorded in 50 *μ*M DCMU-treated cells (Figure 3(b)). This result was consistent with the previous study showing that the highest bidirectional hydrogenase activity of* A. siamensis* TISTR 8012 was obtained when treating cells with 50 *μ*M DCMU under nitrogen deprivation [48]. In this study, it could be explained that the increased hydrogenase activity resulted from a decrease in the chlorophyll fluorescence intensity (Figure 4(b)) and/or the dark respiration rate (Figure 5(b)), indicating that DCMU caused the inhibition of both dark respiration and PSII activity. Our findings that H_2_ production increased after treatment with DCMU agreed with previous studies on cyanobacteria and green algae. In the cyanobacterium* A. cylindrica*, H_2_ production was improved in cells incubated with DCMU, due to the low level of O_2_ [31]. H_2_ photoevolution also increased in cells of a new marine green alga,* P. helgolandica* var.* tsingtaoensis* which were treated with DCMU, as PSII photochemical activity during illumination was completely inhibited by DCMU [22]. The similar result of DCMU inhibition on the photosynthetic electron transport system was reported in the cyanobacteria* Aphanocapsa* 6308 [27],* A. nidulans *[52], and* A. siamensis* TISTR 8012 [48]. In contrast to CCCP result, DCMU treatment did not show an enhanced rate of respiration in* A. halophytica* but showed a significant decrease in respiration rate, especially with high DCMU concentrations (Figure 5(b)), suggesting that DCMU and CCCP possess different functions involved in the respiratory mechanism. Similar results were also found in* A. flos-aquae* [34] and* Chlorella* sp. [53] showing an inhibition of DCMU in respiration rates in the dark.

In the present study, high concentration of CCCP (5 *μ*M) and DCMU (250 *μ*M) induced a significant decrease of H_2_ production (Figure 2) due to the toxicity of CCCP and DCMU to* A. halophytica* cells. These results were confirmed by other experiments showing that too high concentrations of CCCP and DCMU reduced cell and chlorophyll concentrations (Figure 1), the bidirectional hydrogenase activity level (Figure 3), chlorophyll fluorescence intensities (Figure 4), and dark respiration rates (Figure 5).

## 5. Conclusions

Previous studies reported that, due to the limitation of O_2_ on bidirectional hydrogenase activity in the cyanobacterium* A. halophytica*, a very low level of H_2_ was detected after cells were exposed to illumination. In the present study, the well-known photosystem II inhibitors CCCP and DCMU were added to* A. halophytica* samples in an effort to enhance H_2_ production. Both CCCP and DCMU enhanced H_2_ production of* A. halophytica* under light conditions, whereas only CCCP enhanced H_2_ production under darkness. CCCP and DCMU functioned as PSII inhibitors during illumination, resulting in a decrease of chlorophyll fluorescence and O_2_ production in a glass vial. As a result, bidirectional hydrogenase activity was increased and H_2_ production was increased. In addition, CCCP functioned as an uncoupling agent of oxidative phosphorylation, decreasing both proton pumping and ATP synthesis, which resulted in an increase in the respiration rate. This effect helped increase H_2_ production after CCCP treatment under darkness. Our data showed that CCCP can increase H_2_ production by* A*.* halophytica* under both light and dark conditions. However, high concentration and long-term incubation of CCCP led to high cell toxicity. Since* A*.* halophytica *can grow in natural seawater supplemented with 1.76 mM NaNO_3_ [7], it would be useful if this cyanobacterium grown in natural seawater will produce long-term of H_2_ photohydrogen by using PSII inhibitors. This study needs further investigation.

## Data Availability

The data used to support the findings of this study are included within the article.
